# Microscale Diffusiophoresis of Proteins

**DOI:** 10.1021/acs.jpcb.2c04029

**Published:** 2022-10-28

**Authors:** Quentin A. E. Peter, Raphaël P. B. Jacquat, Therese W. Herling, Pavan Kumar Challa, Tadas Kartanas, Tuomas P. J. Knowles

**Affiliations:** † Department of Chemistry, University of Cambridge, Lensfield Road, CB2 1EWCambridge, U.K.; ‡ Cavendish Laboratory, Department of Physics, University of Cambridge, JJ Thomson Avenue, CB3 0HECambridge, U.K.

## Abstract

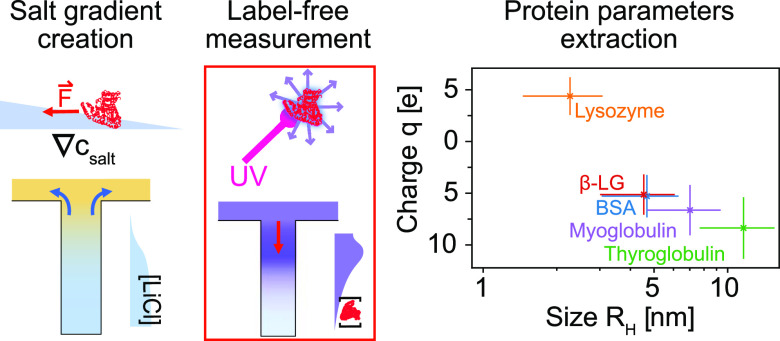

Living systems are characterized by their spatially highly inhomogeneous nature which is susceptible to modify fundamentally the behavior of biomolecular species, including the proteins that underpin biological functionality in cells. Spatial gradients in chemical potential are known to lead to strong transport effects for colloidal particles, but their effect on molecular scale species such as proteins has remained largely unexplored. Here, we improve on existing diffusiophoresis microfluidic technique to measure protein diffusiophoresis in real space. The measurement of proteins is made possible by two ameliorations. First, a label-free microscope is used to suppress label interference. Second, improvements in numerical methods are developed to meet the particular challenges posed by small molecules. We demonstrate that individual proteins can undergo strong diffusiophoretic motion in salt gradients in a manner which is sufficient to overcome diffusion and which leads to dramatic changes in their spatial organization on the scale of a cell. Moreover, we demonstrate that this phenomenon can be used to control the motion of proteins in microfluidic devices. These results open up a path towards a physical understanding of the role of gradients in living systems in the spatial organization of macromolecules and highlight novel routes towards protein sorting applications on device.

## Introduction

Living cells constantly work to remain out of equilibrium, a key requirement for life. One crucial aspect of the non-equilibrium nature of living systems is the ubiquitous presence of gradients in ionic strength, maintained using ion pumps and related molecular machinery. This situation is fundamentally different to the spatially largely homogeneous conditions that characterize protein studies *in vitro* under bulk solution conditions. Understanding the diffusiophoresis of proteins can therefore yield insights in the nature and regulation of protein transport in living organisms.^1^ Although over the past few decades diffusiophoresis has been well studied for larger colloids,^2−8^ little is known about whether single protein molecules, which are much smaller than typical colloids, can also generally undergo diffusiophoresis and which factors can modulate this process. Diffusiophoresis is linked to osmotic processes that take place on the particles surface.^9,10^ Surprisingly, the force applied on the particle does not scale with the surface area^11^ explaining why proteins might undergo non-negligible diffusiophoretic effects, as previously shown for the high mobility protein lysozyme.^12,13^ In biophysics and life sciences, microfluidic techniques are increasingly used to probe the nature of proteins.^14−18^ The rapid growth of the use of microfluidic techniques in research and industry^19,20^ is in part motivated by the fact that, compared to bulk processes, microfluidic processes enable a significant decrease in the required volume of solution. Moreover, under the microfluidic regime it is possible to create laminar flows,^21^ which enable a fine control of dynamic experiments and which allow measurements that are often not possible in bulk solution. Similar phoretic processes, electrophoresis^22,23^ and thermophoresis,^24,25^ are used to develop novel microfluidic techniques in research and industry. Diffusiophoresis is showing promising results for a wide range of applications, from oil recovery^26^ to in-line preconcentration techniques.^27^

In this study, we explore transient protein diffusiophoresis using a microfluidic format, which allows to control protein mass transport by eliminating the influence of factors that are ubiquitous in bulk measurements, such as convection. Previous geometries used for the study of diffusiophoresis of colloids include two channels merging perpendicularly,^28^ parallel flow of two solutions,^29^ diffusion through an hydrogel,^30,31^ or two parallel channels joined by a micro-^32^ or nano-channel.^33^ Here, a simple geometry consisting of a dead-end perpendicular to a main channel is exploited.^34^ Measuring the key properties of proteins such as mobility, size, and isoelectric point is one of the main goals in developing tools for protein science. Diffusiophoresis can be used for sizing colloids and calculating their ζ-potential in microfluidics,^34−36^ and could therefore be extremely useful if this effect proved to be generally significant for proteins. Diffusiophoresis could give additional insight into folding states, as suggested by short polymers simulation.^37^ Here, existing methods are adapted to tackle the unique challenges posed by proteins. First, a label-free UV microscope^38^ is used to measure protein diffusiophoresis in real space. This is widely applicable to proteins and avoids labels that would significantly alter the system properties. Second, new analysis methods are needed as the effect is much weaker than for colloids, and have been developed to extract the relevant biophysical properties of the target protein, such as the hydrodynamic radius and the electrophoretic mobility.

## Methods

### Colloids Diffusiophoresis

In colloids experiments, diffusiophoresis is described as a result of two effects:^2^ chemiophoresis and electrophoresis. The electrophoretic motion is caused by the difference of ionic diffusion coefficients (*D*_±_) of a salt. An electric field (*E*) appears between cations and anions to prevent bulk separation. It depends on the differential ratio of the ion diffusion coefficients (β)^2^
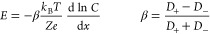
1where *k*_B_*T* is the thermal energy and *Ze* is the ionic charge. The second term, the chemiophoretic contribution, is due to pressure difference inside the double layers of protein,^34^ which is similar to osmosis. One could note that the size of a protein is typically much smaller than the Debye length, so that the applicability of this effect which has been described for large colloids might be doubtful. These two effects are described by the diffusiophoretic mobility of the protein (Γ_p_) that controls the diffusiophoretic speed *u*_p_ = Γ_p_∇ ln *C*. For a purely electrophoretical experiment, the diffusiophoretic mobility is proportional to the ζ-potential of the proteins (ζ_p_)^34,39−41^ and to the electrophoretic mobility (μ_p_)
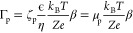
2where ϵ is the permittivity and η is the viscosity of the solvent. The diffusiophoresis therefore depends on the protein through its mobility, and on the salt through the charge of its ions and its β coefficient. The charge (*q*_p_) can be related to the mobility using the Einstein relation
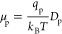
3

More complications could be added to this model. One could think of multidimensional effects such as vortexes caused by diffusioosmosis, but the fast diffusion of proteins and small perpendicular size of the channel decrease these effects. Another limitation of this model is the absence of chemiophoresis and salt concentration dependence of the ζ potential. This limits our ability to accurately determine the charge of the proteins. However, this study can still show a difference of behavior between proteins which is useful for identification and these limitations do not affect the size estimation.

### Experimental Setup

**Figure 1:**
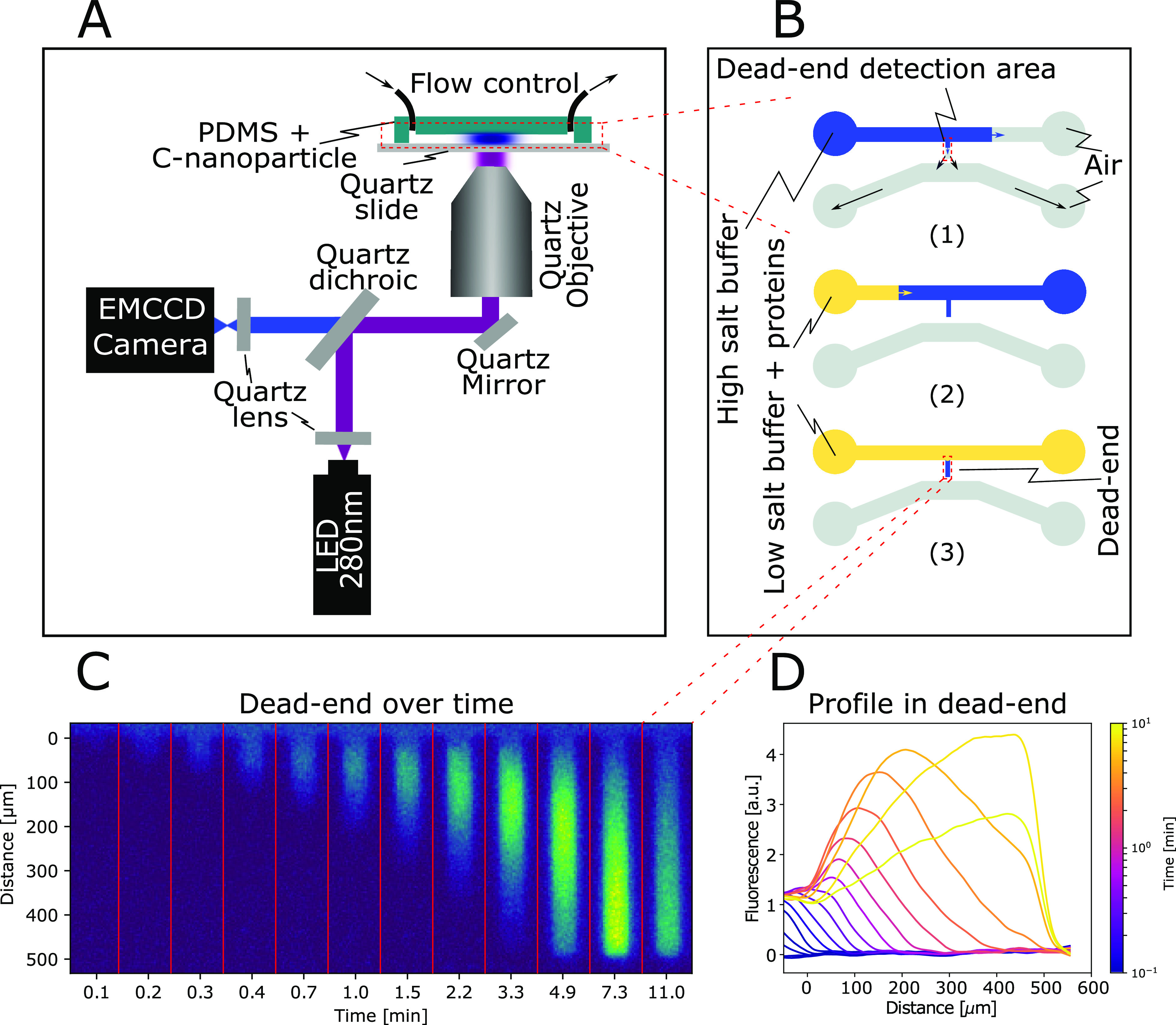
(A) Schematic of the UV label-free microscope used to image a microfluidic device with a dead-end geometry. (B) Microfluidic device design used in this study. (B1) Firstly, the device is filled with a high-salt buffer. The air in the dead-end is pushed through the PDMS in an empty lower channel. (B2) A protein solution is then pushed in the main channel, (B3) leaving only the high salt solution inside the dead-end. (C) A video of the dead-end is taken with logarithmically spaced time points to reduce photobleaching. (D) The channel is detected in each frame and the average over the width is extracted to form a profile.

A microfluidic polydimethylsiloxane (PDMS) device with a dead-end geometry, as shown in Figure 1, is used to create a gradient of salt. It is composed of three regions. The solution is flowed in the main channel, which is a straight channel with a cross section of 500 × 50 μm^2^. The dead-end channel is perpendicular to this channel and its dimensions are 50 × 50 × 500 μm^3^. This is similar to the design presented in Shin *et al.*,^34^ but the design does not use 3D features and is therefore easier to manufacture. Furthermore, a second, unconnected channel is placed near the end of the dead-end to provide an escape to the air going through the PDMS, which is porous. The PDMS is casted on a master created by photo-lithography^42^ and cured. It is then bounded to a quartz slide using a plasma oven.

The device is first filled with the solution which is intended for the dead-end channel. Pressure is applied until the air is evacuated through the PDMS in the second channel. A second solution is then pushed in the main channel, which comes into contact with the first solution at the base of the dead-end channel. The pressure is applied by hand for the priming of the chip and using a neMESYS syringe pump when pushing the protein solution at a flow rate of 600 μL/h.

A UV-LED based microscope is used to detect the proteins autofluorescence at 280 nm.^38^ This is important as a covalently attached label might change the key properties of the protein. In practice, the observed fluorescence intensity at longer times is systematically lower, due to photo-bleaching. To reduce this effect, the images are logarithmically spaced in time.

The proteins have been chosen to represent a wide range of physical parameters. Bovine serum albumin (BSA) is widely used as a protein model. Lysozyme is positively charged. Thyroglobulin is a large protein. β-Lactoglobulin is similar to BSA. Myoglobin is small and has low autofluorescence at the chosen UV-wavelength. The proteins have been purchased from Sigma-Aldrich. The product numbers are: myoglobin from equine skeletal muscle (M0630), thyroglobulin from bovine thyroid (T1001), lysozyme from chicken egg white (L6876), β-lactoglobulin from bovine milk (L3908), and BSA (A7906). The concentrations used are 10 μM for BSA, β-lactoglobulin, and lysozyme, 1 μM for thyroglobulin, and 30 μM for myoglobulin. The experiments are made with three different salts—LiCl, KCl, KIO_3_—at 200 mM. All results can be found online (see Supporting Information).

### Channel Geometry

One might expect that inverting the solute concentration would result in an inversion of the diffusiophoretic velocity. In reality, the diffusiophoretic velocity *u* depends on ∇ ln(*C*) = ∇*C*/*C* where *C* is the solute concentration.^2^ Having the salt in the dead-end causes both the highest gradient and the lowest concentration to be localized at the inlet of the dead-end, therefore leading to the largest effect. In contrast, if the high solute concentration is in the main channel, there are only small guiding fields near the entrance. This greatly reduces the phenomenon.

### Image Analysis

The scripts used for image analysis are available online (see Supporting Information). The images of the dead-end are flattened to remove the non-uniform lightning by fitting a second-order two-dimensional polynomial to the outside of the channel. Detecting the background is only possible if the fluorescence of the proteins is not much higher than the background fluorescence. Otherwise, the fluorescence intensity is used as is. The channel sides are detected by using a Scharr edge detection algorithm. Finally, the intensity is normalized by the median value of the last five frames in the main channel.

The profiles are extracted by taking the average intensity over the center of the width of the channel, ignoring the sides to avoid wall effects. The resulting profiles are then filtered using a repeated Savitzky–Golay filter to reduce noise while conserving the shape. Finally, the profiles are plotted with a different color for each frame time, as shown in Figure 1.

### Finite Elements Simulations

A finite elements software is used to simulate the system (COMSOL Multiphysics 5.2a with microfluidics module and optimization module). In one dimension, a Dirichlet boundary condition is used to fix the protein and salt concentrations at the inlet of the channel, and a Neumann boundary condition is used on the closed end. In two and three dimensions, the main channel is simulated, allowing the main channel flow to enter the dead-end, and allowing a local depletion to occur.

### Fitting

Only profiles with a concentration peak are fitted. As seen on Figure 3, the experimental data fits the profiles well, except in two cases. First, if the peak reaches the end of the channel, the assumption of a semi-infinite channel is clearly broken. Therefore, frames with a significant fluorescence in the last fifth of the channel are not considered. Second, the large intensity difference between the data and the theoretical solution causes the normalized profile to start on a higher level at the channel inlet, as seen on Figure 3. Therefore, the part of the profile between the inlet and the peak is ignored as well. The selected data is illustrated by a solid line, and the ignored data by a dashed line. The solid line is almost completely hidden by the fit.

### Free Flow Electrophoresis and Diffusional Sizing

Free flow electrophoresis is a technique that consists in applying an electric field perpendicularly to the direction of flow and detecting the amount of deviation caused on a stream of particles. The deviation is proportional to the mobility of the particles. Diffusional sizing consists in looking at the diffusion speed under flow and extracting the diffusion coefficient from it. These techniques are used to compare the results with diffusiophoresis.^43,44^

## Results and Discussion

In order to explore whether or not proteins undergo a significant level of diffusiophoresis, we designed a microfluidic device that enables the generation of a localized solute gradient. The experiment is illustrated in Figure 1. After filling the device with a high solute concentration, this geometry allows the content of the main channel to be replaced by a protein solution with low ion concentration, while maintaining the high salt concentration in the dead-end. A strong solute gradient is therefore created at the dead-end inlet. The propagation of proteins over time in this solute gradient is captured by a UV-based autofluorescence microscope enabling label-free measurement of protein concentration.^38^ The data in Figure 1C reveals a significant effect on protein mass transport resulting from this solute gradient.

**Figure 2:**
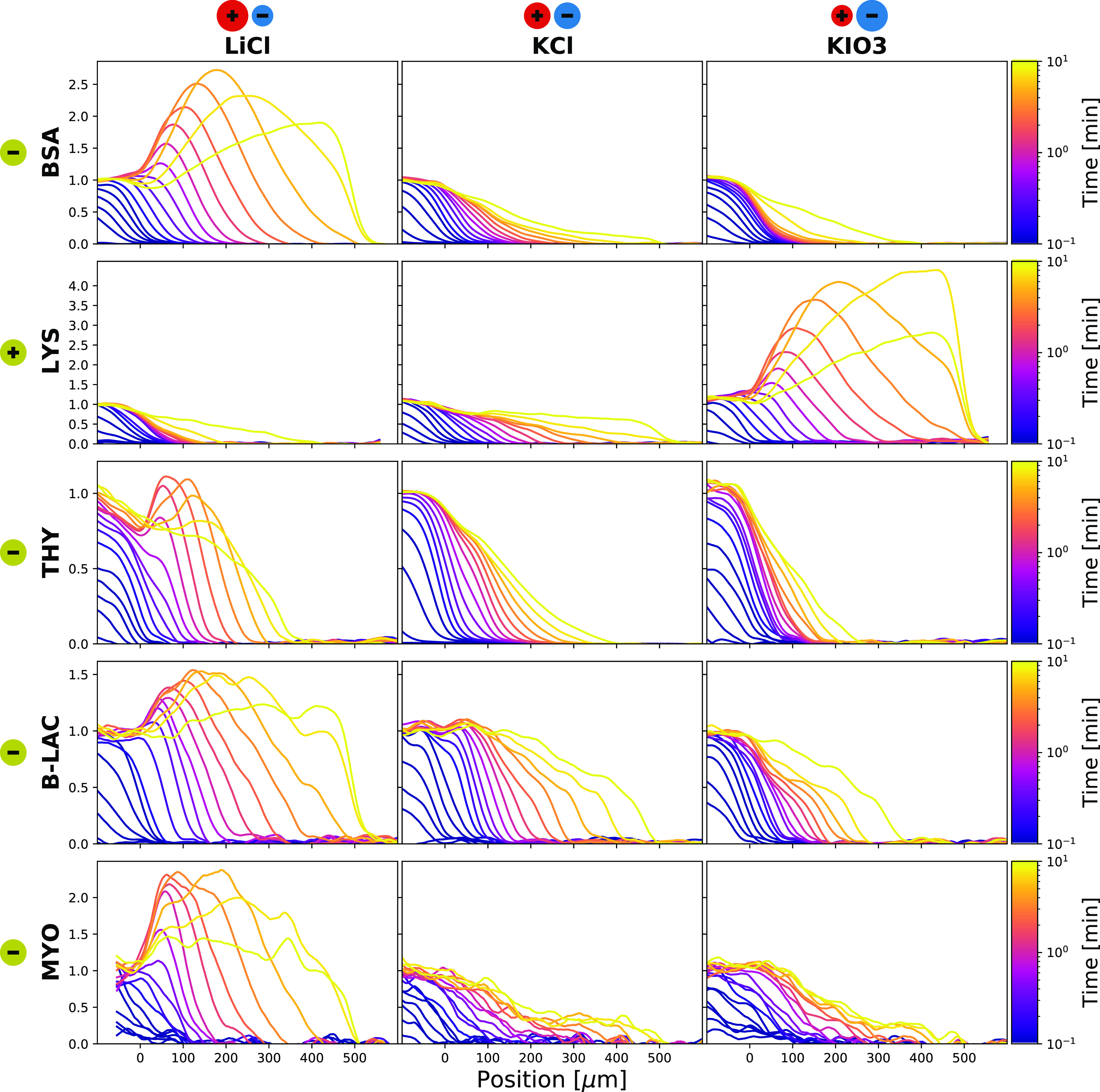
Diffusiophoresis of proteins in different salt gradients. Four negatively charged proteins, BSA (10 μM), thyroglobulin (THY) (1 μM), β-lactoglobulin (B-LAC) (10 μM), and myoglobin (MYO) (30 μM), as well as a positively charged protein, lysozyme (LYS) (10 μM), are placed into a salt gradient. The salts used to create this gradient are lithium chloride (LiCl) (200 mM), potassium chloride (KCl) (200 mM), and potassium iodate (KIO_3_) (200 mM). If the more diffusive salt ion has the same charge as the protein, a concentration peak appears in the channel. When the more diffusive salt ion has the same charge as the protein, the diffusion in the channel is reduced. If the two ions have a similar diffusivity, the effect is small. A sketch of the ions is shown on top of the figure to help visualize the relative diffusivities, where smaller means more diffusive.

In order to understand the origin of this remarkably large diffusiophoretic effect, we consider the key physical parameters governing the motion of large scale objects such as colloids. They exhibit two principal contributions to diffusiophoresis: electrophoresis and chemiophoresis (see the Experimental Section). To investigate whether such effects or other related phenomena play a role for the behavior of proteins whose surface are a factor 10^2^ to 10^3^ smaller than typical colloids, a range of proteins and salts are selected. The importance of the surface area has been discussed in the literature.^11^ In particular, the conjugated salts from strong bases and acids are selected to avoid affecting the pH of the solution. First, we focus on LiCl and KIO_3_, which have a strong difference in the diffusion coefficient of their ions, and then on KCl, whose ions have similar diffusivities. The importance of this strong difference for diffusiophoresis has been described in the literature and is explained in the theory.^9^ This differential behavior is captured by the β coefficient, which is the normalized difference between the ionic diffusion coefficients *D*_+_ and *D*_–_: β ≡ (*D*_+_ – *D*_–_)/(*D*_+_ + *D*_–_). For example, LiCl has *D*_Li^+^_ = 1.03 × 10^–8^ m^2^/s and *D*_Cl^–^_ = 2.03 × 10^–8^ m^2^/s, which gives a normalized difference of β = −0.326. The β coefficient of KIO_3_ (0.298) has roughly the same magnitude and an opposite sign, and KCl (−0.019) has a much smaller magnitude.^45^ Our experiments were designed to test whether the electrophoresis term dominates, resulting in a strong and opposite effect from LiCl and KIO_3_, or if the chemiophoresis term dominates, resulting in a similar effect from all three salts. The results are shown in Figure 2. The first column shows LiCl which dissolves into a more diffusive Cl^–^ anion and a less diffusive Li^+^ cation. An electric field pointing out of the dead-end is created by the difference in ionic diffusion to avoid charge separation. Consistently with the electrophoretic description, BSA, whose charge is negative at pH 7, is attracted and concentrated in the dead-end, forming a visible concentrated peak. The data in the first column of Figure 2 further reveals that lysozyme (LYS), whose charge is positive at pH 7, is by contrast prevented from entering the channel for a few minutes, until the strength of the salt gradient decreases. Next, the effect of KCl whose ions have approximatively the same size in the second column of Figure 2 are investigated. As the profiles are dominated by diffusion, the diffusiophoretic effect is almost negligible. To verify this conclusion, a third salt is tested. KIO_3_ creates a roughly equal and opposite electric field compared with LiCl. As expected, the third column of Figure 2 reveals that BSA diffusion into the dead-end is significantly restricted for several minutes. Lysozyme is instead strongly concentrated and attracted into the dead-end. This result highlights the role of electrostatics and indicates that the electrophoresis is much stronger than the chemiophoretic contribution.

**Figure 3:**
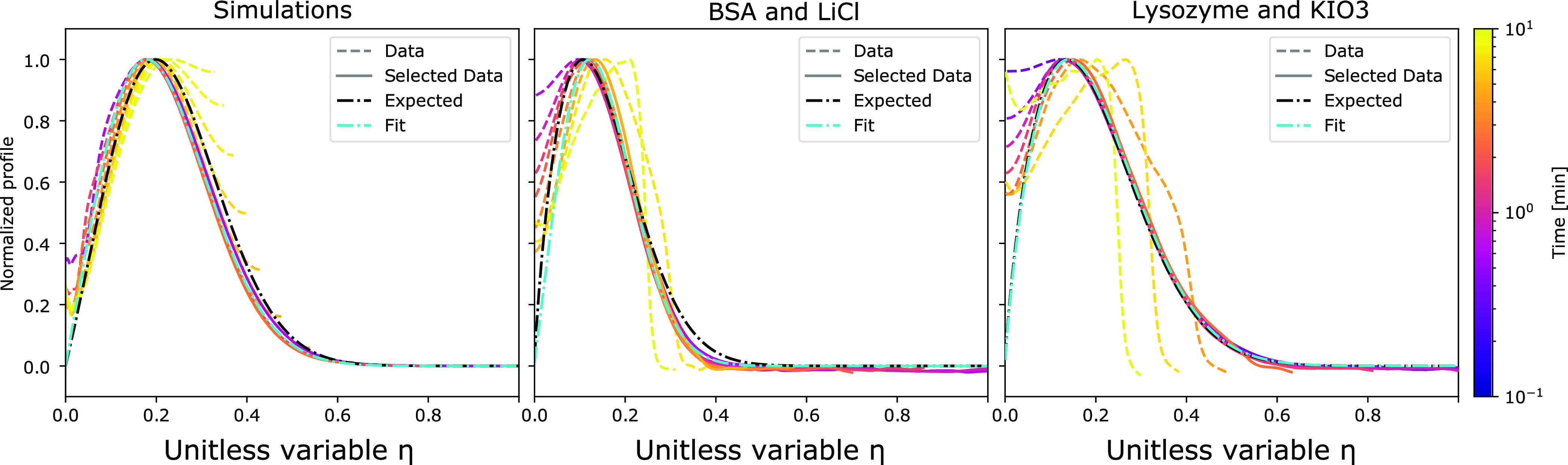
Quantitative description of diffusiophoresis. A finite elements simulation and the two proteins from Figure 2 are plotted as a function of the similarity variable η. The finite elements simulation and the measurements give similar results. Most curves overlap with the 1D model. The differences are explained by two effects. The main channel is not a perfect reservoir. This can be seen on the left near η = 0. The dead-end channel is not semi-infinite. This is highlighted by the later, yellow curves not overlapping when reaching the end of the channel. Both the fitted profile (cyan) and the expected profile (black) are a good match to the measured data. The simulation is a 2D simulation with protein diffusion coefficient of 5.9 × 10^–11^ m^2^/s and diffusiophoresis coefficient of −1.5 × 10^–10^ m^2^/s. The corresponding expected profile is the solution of eq 4 for the same values. For BSA and lysozyme plots, the expected profiles are the solution to the same equation with electrophoretic mobilities measured by free flow electrophoresis^46^ of respectively −0.988 × 10^–8^ and 1.76 × 10^–8^ m^2^/V s, and hydrodynamic radii measured by diffusional sizing^47^ of respectively 3.48 and 2.05 nm.

**Figure 4:**
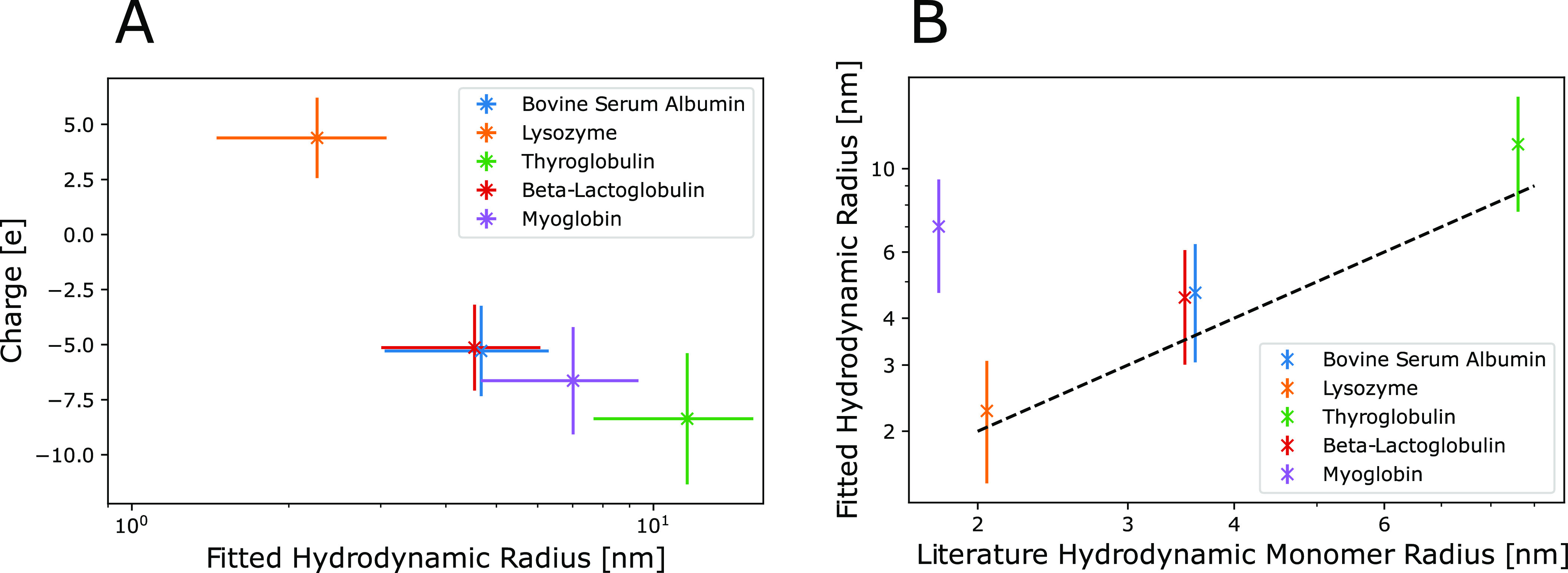
Fitting results for the five proteins from Figure 2. The error bars are estimated from the fitting. The hydrodynamic radius is extracted from the fitted diffusion coefficient. The charge is extracted from both the diffusion and the diffusiophoresis coefficients, by making the assumption that the system is dominated by the electrophoretic contribution to diffusiophoresis. The relative error is about 34% on the radius and 38% on the charge. (A) Graph highlighting the separation potential of using two dimensional data. Proteins that would be indistinguishable by either charge or hydrodynamic radius can be distinguished. (B) Comparison of data with literature monomer hydrodynamic radius.^49−52^ While most proteins match their monomeric size, myoglobin is likely aggregating under the current conditions.

A concentration peak becomes visible when the charge sign of the protein matches the more diffuse salt ion. The position of the peak depends mostly on the diffusiophoresis strength, and the width of the peak depends on the protein size. This opens up the possibility to fit the peaks to extract this information about the proteins. The physics can be captured in one dimensional space by introducing a similarity variable,^48^, describing distances *x* relative to the mean diffusional distance of the salt with diffusion coefficient *D*_s_ at a time *t*. The protein concentration (*N*) depends on the diffusion (*D*_p_ d^2^*N*/dη^2^) and on a driving force from the gradient in the channel potential of the salt creating a dimensionless velocity (dln(*C*/*C*_main_)/dη)
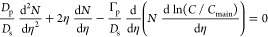
4The dimensionless constants represent the ratio of the protein diffusion coefficient (*D*_p_) and diffusiophoretic coefficient (Γ_p_) with the salt diffusion coefficient (*D*_s_). The salt diffusion coefficient captures the diffusion of both ions. This results in a weighted average of the ionic diffusivities. The diffusiophoretic coefficient depends on the protein and on the salt properties. In the dilute limit, the salt concentration *C* is given by a single constant (0 ≤ α ≤ 1), which is the ratio of the concentration in the main channel (*C*_main_) by the initial concentration in the dead-end
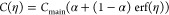
5Interestingly, this simple one-dimensional analysis predicts qualitatively the observed trends, thus capturing the essential physics, as shown in Figure 3. The influence of adsorption at the timescale of this experiment is considered as limited in our analysis notably due to photobleaching. A quantitative comparison reveals that the predicted peak heights are higher than those observed in the experiments. To understand the origin of this effect, finite elements simulations for multidimensional systems are next performed. The reason for the observed differences in the peak heights is a depletion of analytes at the inlet of the dead-end that is not captured in the one dimensional analysis. However, when normalized by the height of the concentration peak, the profiles have the same shape, as illustrated in Figure 3. The cyan profiles, fitted with eq 4, match well the measurements and the finite elements simulations. The width of the peak is directly related to the protein diffusion coefficient, and the protein mobility can be obtained from the location of the peak. The black profiles in Figure 3 indicate the expected curves from the mobility and radius measured by free flow electrophoresis^46^ and diffusional sizing.^47^ The same fitting is applied to the other protein experiments and the results are shown in Figure 4. The lysozyme protein has the largest mobility, explaining why the effect is the strongest among tested proteins. The myoglobin size is much larger than anticipated, which indicates that aggregation is likely taking place. This could be partly caused by the high myoglobin concentration used, to compensate for the low autofluorescence at the selected UV-wavelength.

The finite elements simulations are validated by experiments. Moreover, they provide us with an opportunity to query which experimental parameters can be optimized for future developments to maximize the strength of the diffusiophoretic effect. For example, these simulations show how the concentration power depends on the salt properties. Figure S4 in the Supporting Information summarizes the strength of the effect while different experimental parameters are varied. The most impactful parameter is the diffusiophoresis coefficient, which can be maximized by choosing a salt with a large difference in ionic diffusion coefficient. As explained in the Supporting Information, this is achieved by maximizing the difference in ionic hydrodynamic radii. Other optimizations include a small diffusion coefficient for the salt and a salt concentration difference of at least 2 orders of magnitudes between the main and side channels.

## Conclusion

This paper describes a direct, real-space observation of diffusiophoresis of proteins. This spatial effect is commonly ignored in descriptions of biological systems. In this context, a method to measure protein diffusiophoresis could open the way to novel physiological discoveries. Diffusiophoresis, which is dependent on particle size, is significant for proteins and could have applications for the manipulation of proteins in microfluidic devices. In our diffusiophoretic experiments, proteins could be concentrated by up to a factor of 4. Moreover, these proteins could be prevented from entering the channel during several minutes. The protein diffusion and diffusiophoretic coefficients were estimated. We could show that the electrophoretic contribution to diffusiophoresis is much more consequential than the chemiophoretic contribution. Finally, we discussed how to increase the effect by choosing a salt that is composed of ions with a large difference in relative diffusion coefficient. This opens up the door to fundamentally new microfluidic approaches for protein detection and characterization.

## Supporting Information Available

SI_microscale_diffusiophoresis_proteins.pdf: Supporting Information for “microscale diffusiophoresis of proteins”. Polyvalent ions: mathematical derivation of the polyvalent ion case. COMSOL simulations: simulations to explore the diffusiophoretic effect. Experiments: replotting of the experimental results with the similarity variable, comparison with diffusion, NaOH experiment. Fits: fitting of simulations to check pipeline. https://doi.org/10.17863/CAM.48786: all raw data and simulations. https://doi.org/10.5281/zenodo.3636078: data analysis scripts (PDF)
