# Supplementary material for: Microscale Diffusiophoresis of Proteins
Source: J Phys Chem B. 2022 Oct 28;126(44):8913–20. doi: 10.1021/acs.jpcb.2c04029 (PMC9661530; doi:10.1021/acs.jpcb.2c04029)
Supplement: Supplementary file 1 — jp2c04029_si_001.pdf [file jp2c04029_si_001.pdf]

Supplementary Information for "Microscale Diffusiophoresis of Proteins"

Quentin A. E. Peter,[†,¶] Raphaël P. B. Jacquat,[‡,¶] Therese W. Herling,[†] Pavan Kumar Challa,[†] Tadas Kartanas,[†] and Tuomas P. J. Knowles[*,†]

†*Department of Chemistry, University of Cambridge, Lensfield Road, CB2 1EW, Cambridge, UK.*

‡*Cavendish Laboratory, Department of Physics, University of Cambridge, JJ Thomson Avenue, CB3 0HE, Cambridge, UK.*

¶*These authors contributed equally to this work.*

E-mail: tpjk2@cam.ac.uk

Polyvalent Ions

The description of diffusiophoresis in the literature for electrophoresis based diffusiophoresis usually depicts a salt whose two ions have the same valence or charge number ($Z_\pm$).[1–4] This is not always the case, especially if - to optimise the β parameter - one uses a large and highly charged molecule with many counter-ions. Here, the general case of ions polyvalence is derived. An electric field is assumed to prevent the charge density from being non-zero. The diffusion of small ions is slowed down, while the diffusion of larger ions is accelerated by this field. As the density of charge is assumed to be zero, $\vec{\nabla} \cdot \vec{E} = 0$ from Maxwell equations. Each ionic specie i has a concentration c_i, a diffusion coefficient D_i, and a mobility μ_i. The motion is controlled by the convection-diffusion equation:

$$\frac{\partial c_i}{\partial t} = D_i \nabla^2 c_i - \mu_i \vec{E} \cdot \vec{\nabla} c_i$$

The mobility μ_i depends on the charge and on the diffusion coefficient:

$$\mu_i = \frac{D_i q_i}{k_B T} \tag{1}$$

The condition of charge neutrality can be written as $\sum c_i q_i = 0$. Taking the derivative with respect to time and integrating over space leads to a formula for the electric field needed to avoid charge separation:

$$\vec{E} = k_B T \frac{\sum q_i D_i \vec{\nabla} c_i}{\sum q_i^2 D_i c_i} \tag{2}$$

This is further simplified in the case of a single anion and a single cation with charge $q_\pm = \pm Z_\pm e$, where e is the elementary charge. In this case, the concentration of ions is proportional to the salt concentration c:

$$\vec{E} = \frac{D_+ - D_-}{D_+ Z_+ + D_- Z_-} \frac{k_B T}{e} \vec{\nabla} \ln c \tag{3}$$

This leads to the definition of a new unit-less parameter β_Z that uniquely describes the salt contribution to the diffusiophoresis.

$$\beta_z = \frac{D_+ - D_-}{D_+ Z_+ + D_- Z_-} \tag{4}$$

If the ions have the same valence ($Z_+ = Z_-$), the usual result is recovered, and $\beta_Z = \beta/Z$. The diffusiophoretic coefficient therefore only depends on the protein mobility μ_p and on the salt β_Z coefficient:

$$\Gamma_p = \frac{k_B T}{e} \mu_p \beta_Z \tag{5}$$

A similar derivation can be used for the salt diffusion coefficient D_s:

$$D_s = D_+ D_- \frac{Z_+ + Z_-}{D_+ Z_+ + D_- Z_-} \tag{6}$$

From the main text, the unitless coefficient that controls diffusiophoresis is Γ_p / D_s:

$$\frac{\Gamma_p}{D_s} \propto \frac{1}{Z_+ + Z_-} \left(\frac{1}{D_-} - \frac{1}{D_+} \right) \tag{7}$$

The inverse of the diffusion coefficient is proportional to the hydrodynamic radius. Therefore, maximising the diffusiophoretic effect corresponds to maximising the difference between the ionic hydrodynamic radii.

COMSOL Simulations

The theoretical solution for the semi-infinite channel predicts a concentration that is much higher than what is seen in the experiments. To understand this discrepancy, COMSOL simulations are done in one, two, and three dimensions. In one dimension, the salt and protein concentrations are fixed at the dead-end inlet. In two and three dimensions, the main channel and the flow are simulated. An example of the protein distribution is shown in

Figure (S1). A half circle can be seen in two and three dimensions, where the main channel flow penetrates in the dead-end. While the intensity of the one dimensional simulation is very close to the intensity predicted by the theoretical solution, as shown in Figure (S2), the intensities of the higher dimensional simulations are much lower. However, the solution seems to fit perfectly when the profiles are normalised. In two and three dimensions, a small offset can be seen. It leads to a small underestimation of the diffusiophoresis coefficient, as discussed below.

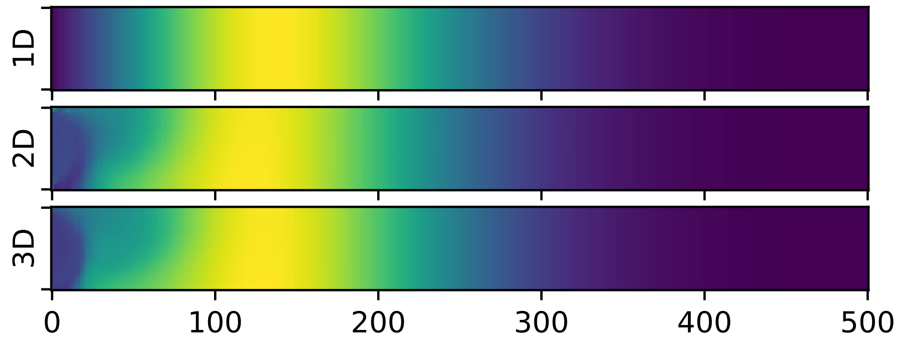

Figure S1: Spatial distribution of the concentration in the channel for the COMSOL Figures after two minutes. The colourmaps are normalised for each plot, so that the intensities cannot be compared here.

Figure (S3) shows the effect of varying several parameters on the strength of the diffusiophoresis effect. First, the effect of the salt concentration ratio between the main channel and the initial dead-end concentration are investigated, in Figure (S3A). As expected, a ratio of one does not lead to any diffusiophoretic effect. As the ratio decreases, the effect becomes stronger but rapidly saturates when the main channel molarity is two to three orders of magnitude below the dead-end molarity. Further, a protein with a large diffusion coefficient is unsurprisingly harder to concentrate (Figure S3B). As expected, the diffusiophoresis coefficient is a major contributor to the concentration (Figure S3C). Therefore, to see a large effect, β should be maximised. A perhaps less intuitive result is shown in the final plot (Figure S3D). As the salt diffusion coefficient increases, the intensity decreases. This is explained in the main text in Equation (1), as the unit-less parameter that controls

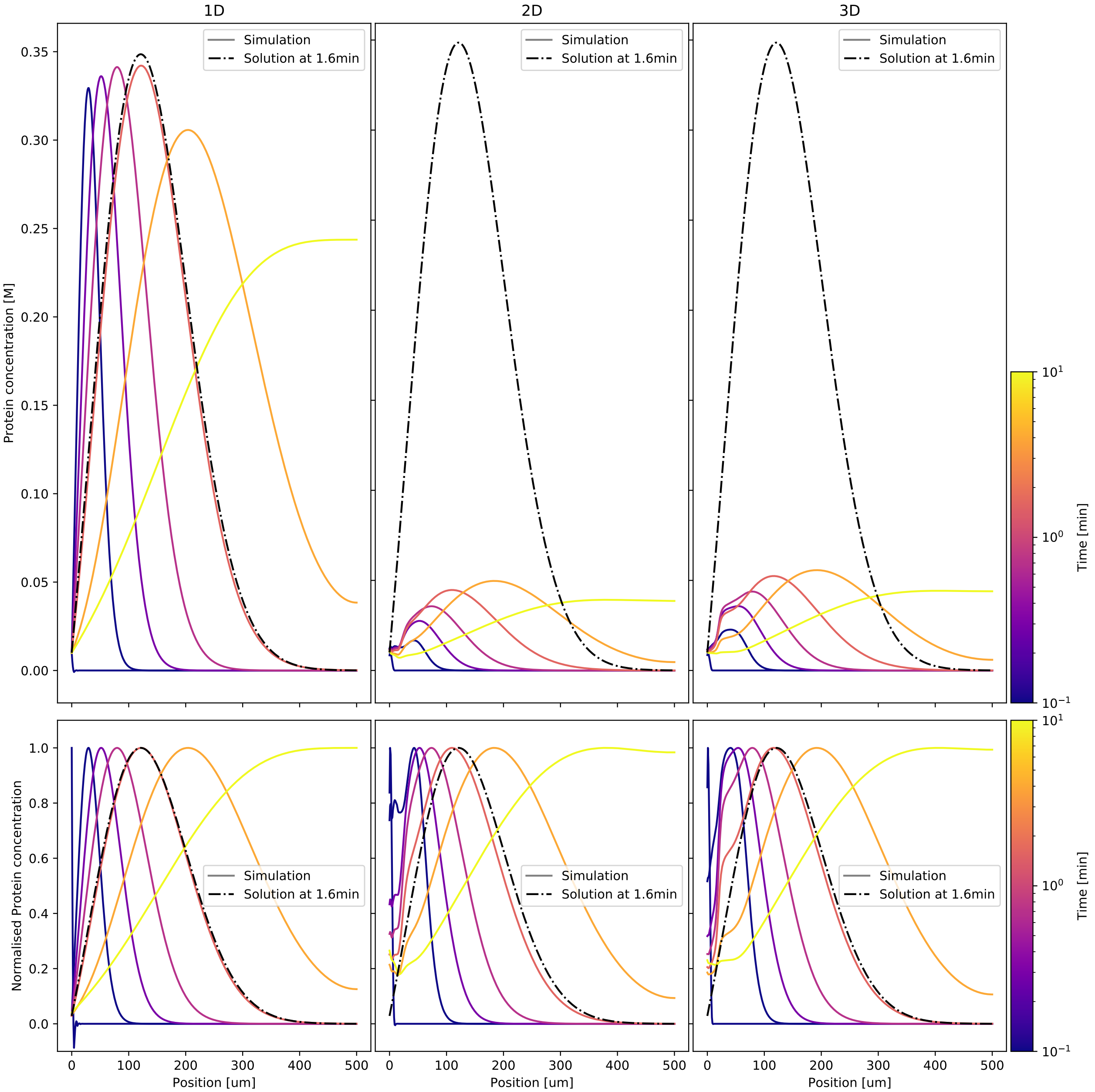

Figure S2: Comparison of one, two, and three dimensional simulations. The intensity of the partial differential equation and of the one dimensional simulation are similar and much larger than the intensity of two and three dimensional simulations. When normalised, the three simulations are almost indistinguishable.

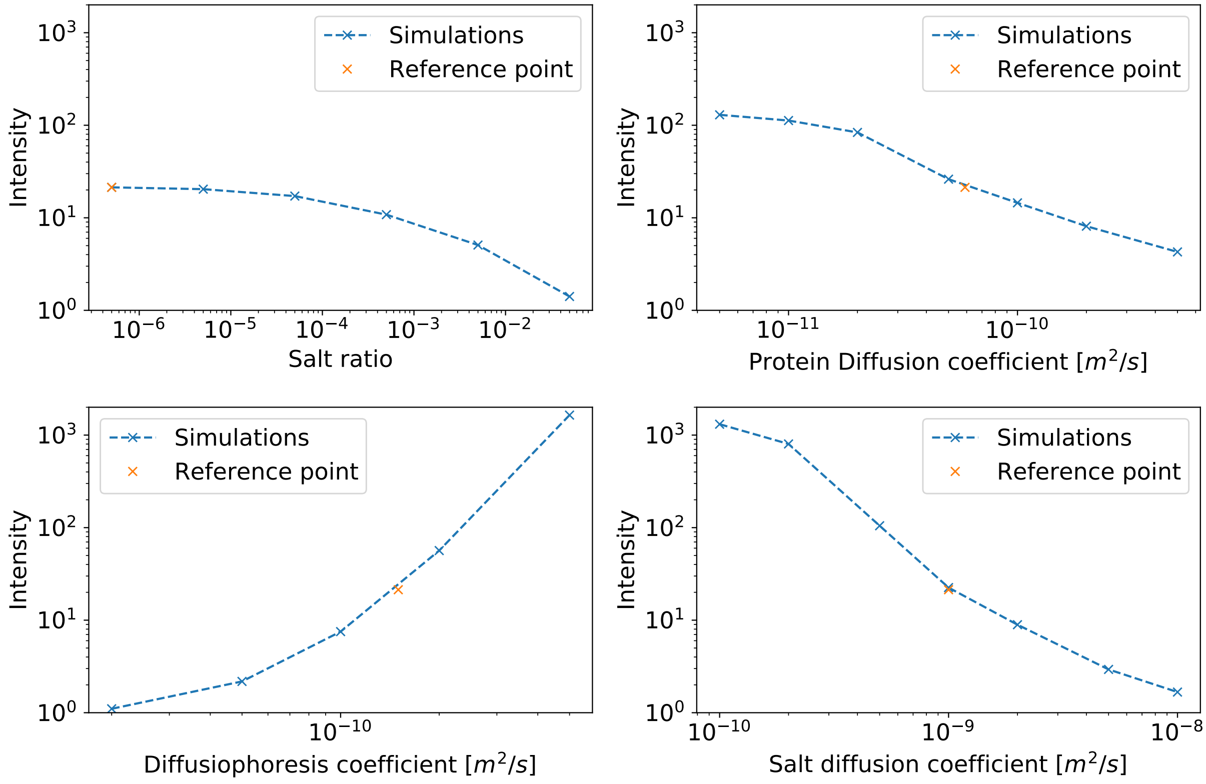

Figure S3: Role of the physical properties of proteins on the strength of the diffusiophoresis effect. The maximum concentration of the simulations is shown as a function of (A) salt ratio, (B) protein diffusion coefficient, (C) diffusiophoresis coefficient, and (D) salt diffusion coefficient. The intensity is the ratio of the maximal concentration with the initial protein concentration. The reference point is the same between the four graphs.

the diffusiophoresis is Γ_p/D_s. An experiment designed to analyse proteins using the diffusiophoretic effect should therefore use a high concentration of a salt that has a large difference in ionic diffusion coefficient, and whose own diffusion coefficient is small.

Experiments

Plotting the experiments against the similarity variable η can give a better vision of whether or not diffusiophoresis is taking place. This is shown in Figure (S4). The small diffusiophoretic effect of KCl, while nowhere as large as the LiCl and KIO_3 versions, can be observed. The comparison with diffusion can be seen in Figure (S5), where the salt gradient is removed by placing salt both in the dead-end and in the main channel with the proteins. The shape of the similarity plot therefore matches the pure diffusion prediction (dashed green line).

The salt concentration ratio is an important parameter as it limits the concentration of buffer that can be used with the proteins. Even using proteins without buffer can significantly decrease the diffusiophoretic strength. BSA is itself an ionic molecule, and is accompanied by many counter-ions. The molecular weight of BSA is typically three orders of magnitude higher than a salt such as LiCl. Assuming that the concentration of counter-ions is a thousand times higher than the protein concentration, Figure (S6) is consistent with the simulation results discussed in the main text: decreasing the concentration from 1000 μM to 100 μM results in a peak ten times higher, while the next decrease of an order of magnitude only increases the peak height by 30%. The concentration of any salt or counter-ions should therefore be at least two orders of magnitude lower than the dead-end salt concentration. The next test consists in verifying if the diffusiophoresis coefficient dependence is really as strong as Figure (S3) seems to indicate. To that end, NaOH is compared with LiCl, as the β coefficient of NaOH is twice as large as the one of LiCl. To avoid a large effect from the massive change in pH, thyroglobulin, whose electro-phoretical mobility is roughly constant

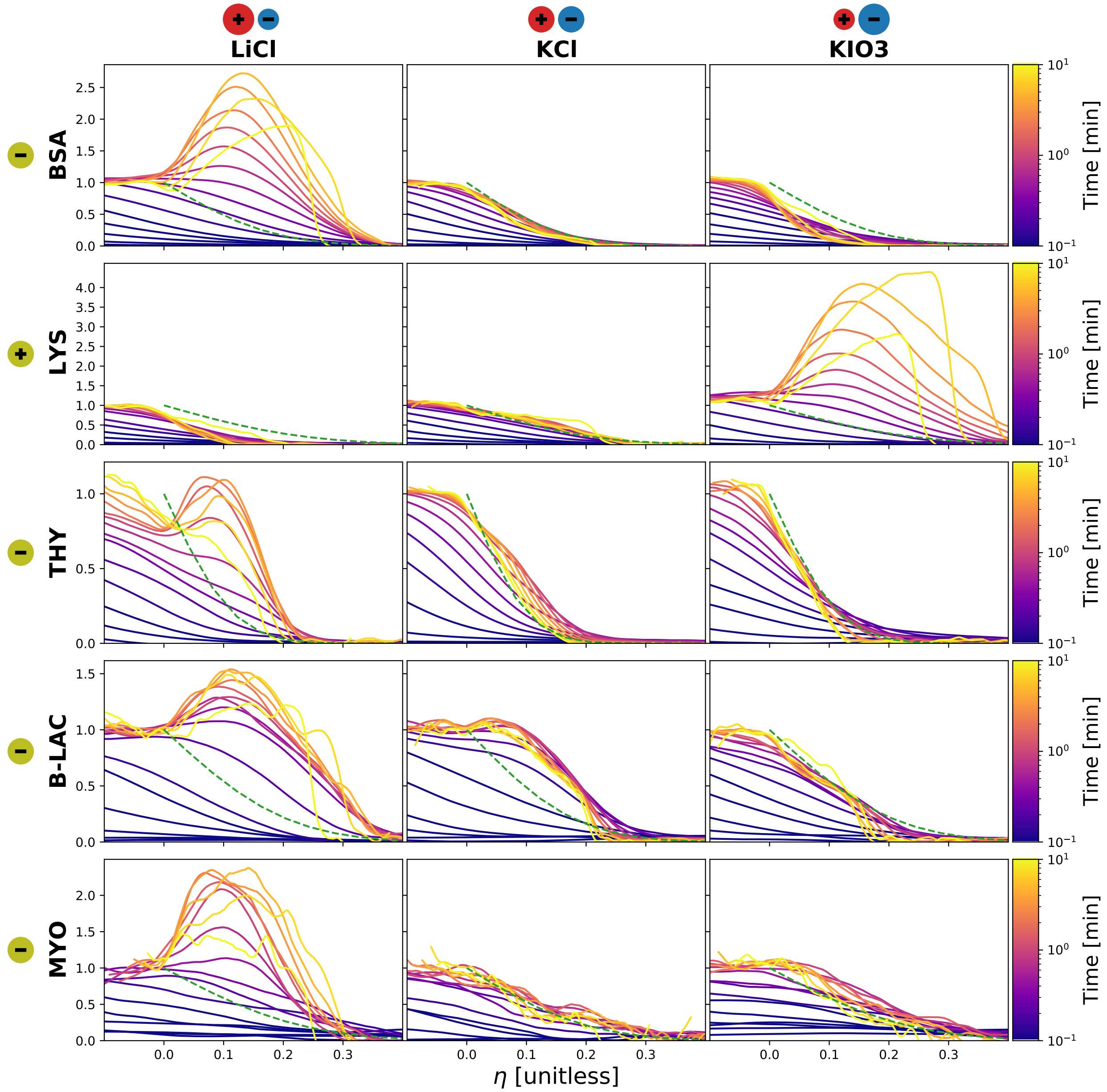

Figure S4: Diffusiophoresis of proteins in salt gradient versus the similarity variable η. Four negatively charged proteins, Bovine Serum Albumin (BSA) (10 uM), Thyroglobulin (THY) (1 uM), Beta-Lactoglobulin ($\mathrm{B-LAC}$) (10 uM), and Myoglobin (MYO) (30 uM), as well as a positively charged protein, Lysozyme (LYS) (10 uM), are placed into a salt gradient. The salts used to create this gradient are Lithium chloride (LiCl) (200 mM), Potassium chloride (KCl) (200 mM), and Potassium iodate (KIO_3) (200 mM). If the smaller salt ion has the same charge as the protein, a concentration peak appears in the channel. If the larger salt ion has the same charge as the protein, the diffusion in the channel is reduced. If the two ions have a similar charge, no effect is visible. The green dotted line shows the expected profile from diffusion alone, which should be the same at any time when plotting against η.

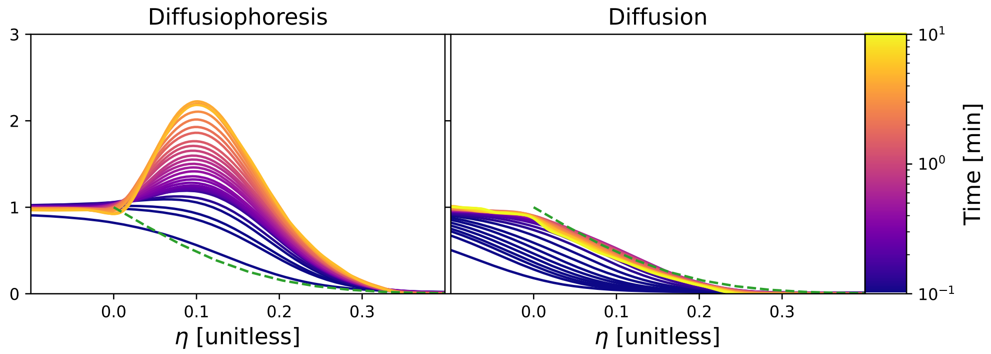

Figure S5: Diffusiophoresis and pure diffusion of BSA. For diffusiophoresis, 10 uM of Bovine Serum Albumin is placed in the main channel and 200 mM of Lithium chloride in the dead end, creating a salt gradient. For diffusion, 200 mM of Lithium chloride is further added in the main channel to flatten the gradient and remove the diffusiophoretic effect. In the latter case, the data matches the green dotted line, representing the expected shape for diffusion.

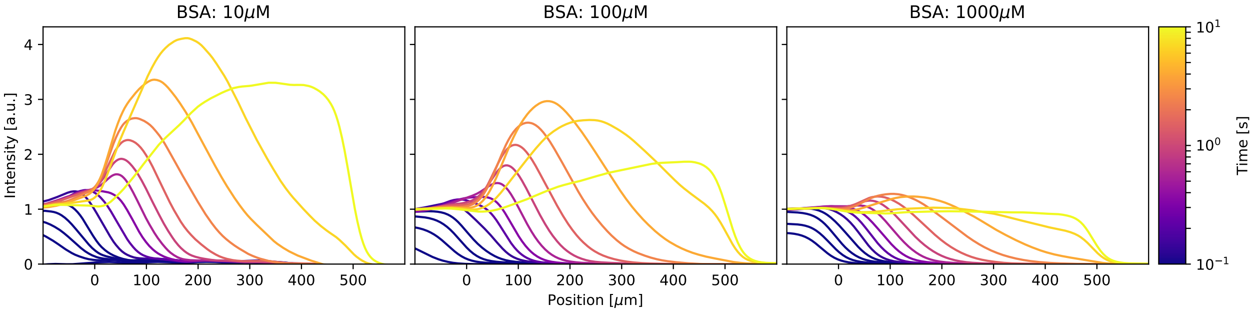

Figure S6: Effect of protein concentration on diffusiophoresis for BSA. The salt is 2 M LiCl.

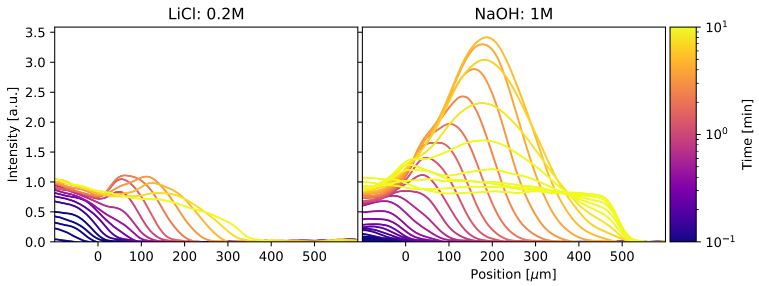

Figure S7: Effect of diffusiophoretic coefficient on 1 uM Thyroglobulin. The salts are 0.2 M LiCl and 1 M NaOH.

for pH of 7 and above,[5] is selected. As shown in Figure (S7), the peak is 16 times higher when simply doubling the diffusiophoretic coefficient.

Fits

The simulations show a good agreement with the theoretical solutions after normalisation. Therefore, fitting the profiles appears to be a promising way of extracting information about the proteins. To validate this approach, the simulated profiles are fitted with the theoretical solution. As the theoretical solution assumes a semi-infinite channel, the profiles with significant intensity in the last fifth of the channel are excluded. Similarly, the part of the profiles between the main channel and the peak are not fitted, as the intensity difference changes this part of the profiles. As can be seen in Figure (S2), a slight offset in the fitted diffusiophoresis coefficient and a good agreement with the diffusion coefficient are expected. This is indeed what is shown in Figure (S8). The error on the diffusion coefficient is consistently smaller than 10% and the error on the diffusiophoresis appears to have a constant offset of -25%. A closer look at a few simulations leads to a better understanding of these graphs. The diffusiophoresis coefficient seems to increase slightly with the salt ratio, as shown in Figure (S8A). For ratios close to one, the concentration is much lower, as shown in Figure (S9A). The fitted coefficient increases when the diffusiophoretic effect disappears. The following simulations are done with the lowest channel salt concentration. In Figure (S8B), the fitted diffusion becomes much higher when the diffusion coefficient is low. This is unsurprising as the slope becomes really steep and does not change much, as shown in Figure (S9B). On the one hand, effects such as numerical diffusion could easily explain this change in fitted diffusion coefficient. On the other hand, the diffusiophoresis coefficient appears to be roughly constant. In Figure (S8C), the diffusion coefficient is quite stable when varying the diffusiophoresis coefficient. However, the fitted diffusiophoresis coefficient itself shows a relative increase for lower values of the simulated diffusiophoresis coefficient. As shown in

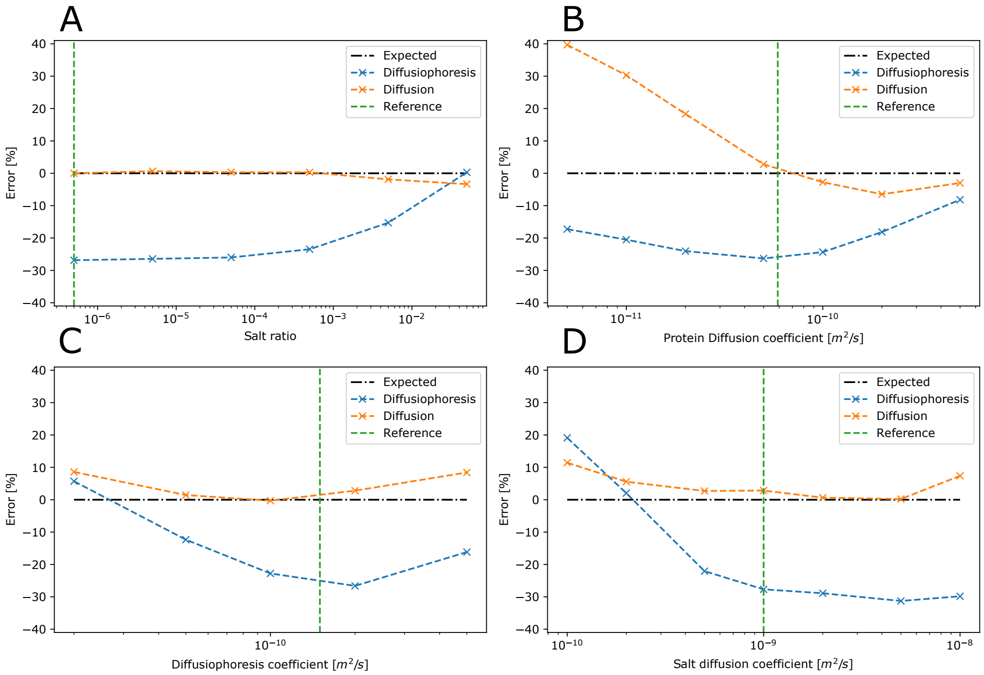

Figure S8: Error in the fit of the simulations. The blue lines represent the error on the diffusiophoresis coefficient. The orange lines represent the error on the diffusion coefficient. The reference value is the same between the four graphs.

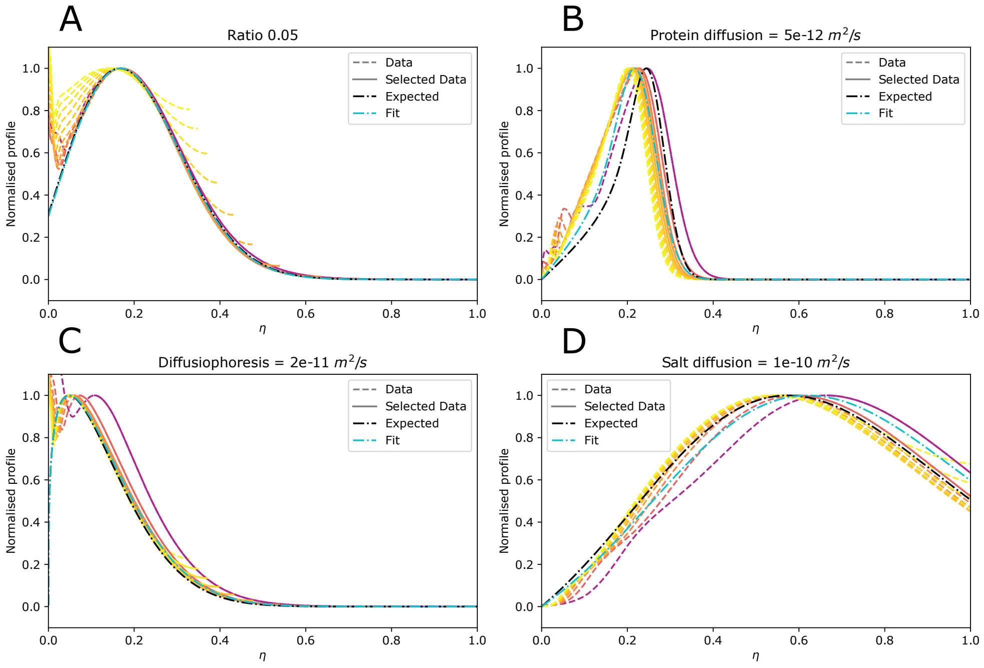

Figure S9: Selected simulation profiles and fits.

Figure (S9C), this could be explained by the simulation taking more time to converge to the theoretical solution. This problem is even more apparent in Figure (S8D), where the fitted diffusiophoresis appears to be too large for low salt diffusion. Looking at Figure (S9D), the problem appears: only the first time points do not have a significant fluorescence at the end of the channel. Unfortunately, these time points are taken before the simulation is converged to the theoretical solution.

Table S1: Values of different β coefficients at 25°C. Values taken from[6,7] for LiCl, CsCl and KI0$_3$; and from[8] for NaCl and KCl.

Salt	NaOH	LiCl	NaCl	KCl	CsCl	KIO_3	HCl
β	-0.597	-0.326	0.207	-0.019	0.005	0.298	0.642

References

(1) Salgin, S.; Salgin, U.; Bahadir, S. Zeta potentials and isoelectric points of biomolecules: the effects of ion types and ionic strengths. *Int J Electrochem Sci* **2012**, *7*, 12404–12414.

(2) Shin, S.; Um, E.; Sabass, B.; Ault, J. T.; Rahimi, M.; Warren, P. B.; Stone, H. A. Size-dependent control of colloid transport via solute gradients in dead-end channels. *Proceedings of the National Academy of Sciences* **2016**, *113*, 257–261.

(3) Prieve, D. C.; Roman, R. Diffusiophoresis of a rigid sphere through a viscous electrolyte solution. *Journal of the Chemical Society, Faraday Transactions 2: Molecular and Chemical Physics* **1987**, *83*, 1287–1306.

(4) Prieve, D.; Anderson, J.; Ebel, J.; Lowell, M. Motion of a particle generated by chemical gradients. Part 2. Electrolytes. *Journal of Fluid Mechanics* **1984**, *148*, 247–269.

(5) Ui, N. Electrophoretic mobility and isoelectric point of hog thyroglobulin. *Biochimica et Biophysica Acta (BBA)-Protein Structure* **1972**, *257*, 350–364.

(6) Goswami, A.; Acharya, A.; Pandey, A. Study of self-diffusion of monovalent and divalent cations in Nafion-117 ion-exchange membrane. *The Journal of Physical Chemistry B* **2001**, *105*, 9196–9201.

(7) Yuan-Hui, L.; Gregory, S. Diffusion of ions in sea water and in deep-sea sediments. *Geochimica et Cosmochimica Acta* **1974**, *38*, 703–714.

(8) Velegol, D.; Garg, A.; Guha, R.; Kar, A.; Kumar, M. Origins of concentration gradients for diffusiophoresis. *Soft Matter* **2016**, *12*, 4686–4703.
